# Phage display screening identifies a novel peptide to suppress ovarian cancer cells *in vitro* and *in vivo* in mouse models

**DOI:** 10.1186/s12885-015-1891-8

**Published:** 2015-11-10

**Authors:** Cong Zhou, Jiali Kang, Xiaoxia Wang, Wei Wei, Wenyan Jiang

**Affiliations:** Department of Obstetrics and Gynecology, Maternity and Children’s Healthcare Hospital of Foshan, Foshan, 528000 Guangdong China; Department of Obstetrics and Gynecology, Guangzhou First People’s Hospital, Guangzhou Medical University, Guangzhou, 510180 China; Foshan Hospital of TCM, Foushan, 52800 China

**Keywords:** Phage display, Peptide, Ovarian cancer, Tumor cell viability, Invasion, Adhesion, Nude mouse tumor model

## Abstract

**Background:**

Ovarian cancer is a possibly lethal gynecological malignancy and this study utilized phage display technology to screen and identify peptides that specifically bind to ovarian cancer cells and explored the effects of these peptides on ovarian cancer cells *in vitro* and *in vivo*.

**Methods:**

The phage displayed peptide library was used to isolate the peptides binding to and internalizing into the ovarian carcinoma cells. Positive phage clones were characterized with DNA sequencing and bioinformatics analysis and then validated with immunofluorescence. Subsequently, the selected peptides were investigated for their cancer-related functions, including cell adhesion, spreading, motility, and invasion *in vitro* and *in vivo*.

**Results:**

Peptide1 read as SWQIGGNwas the positive peptide and showed preferential binding to the target cells. Peptide 1 also inhibited cell proliferation, migration, invasion and adhesion of ovarian cancer HO8910 cells *in vitro. In vivo*, Peptide 1 led to a lower tumorigenicity of HO8910 cells, which was characterized by the inhibitory effect on tumor growth and metastasis of ovarian cells.

**Conclusion:**

These studies demonstrate that the phage display-identified tumor cell-binding peptide was able to control ovarian cancer cell viability, migration, invasion, and adhesion capacity *in vitro* as well as tumor growth and metastasis *in vivo*. Future studies will be aimed at evaluating the clinical efficacy of the peptide SWQIGGN in ovarian cancer patients.

## Background

Ovarian cancer is the most lethal gynecological malignancy and is characterized by insidious onset, rapid progression and poor survival [1, 2]. Indeed, most of ovarian cancer patients are diagnosed at an advanced stage of the disease with evidence of metastasis spreading beyond the ovaries [3] and the relapse rate of early stage ovarian cancer is up to 40 % [4–7]. To date, the treatment of ovarian cancer is much like those of all other cancers and includes surgery, chemotherapy, radiation therapy and immunotherapy [8]. If the tumor is still confined in the ovary, surgery could be used to cure, while, for decades, paclitaxel and carboplatin chemotherapy have been used as the general standard of care for most ovarian cancer patients. However, tumor resistance does develop and certain subtypes of ovarian cancer are insensitive to chemotherapy. Radiation therapy is not effective for advanced stages of this disease and immunotherapy is just in the early stages of clinical trials [9]. Therefore, the identification of novel targets is urgently needed to develop innovative agents to effectively control ovarian cancer metastasis and progression [10]. To this end, recent studies reported that the secretion of cell surface proteins in human cancer cells could be especially important as therapeutic targets [11–13]. Our research is focused on identifying novel peptide-based probes using combinatorial chemistry and bacteriophage (phage) display to facilitate and identify novel cell surface molecules that could serve as biomarkers and therapeutic targets. As phage display is a useful technique we are able to examine protein–protein, protein–peptide, and protein–DNA interactions [14–16]. The “displayed” (selected) peptide is used to identify the receptor on the cell surface, which could be further used as a diagnostic marker or therapeutic target [14, 17]. Previous studies utilized phage display peptide libraries to isolate and identify peptides that not only specifically bind to cell receptors in a cell-specific manner, but also exerted biological effects on the target cells [18–21]. Thus, we aimed to screen and identify peptides that could possess highly specific binding capabilities to human ovarian cancer cells, and then to investigate the potential of these selected peptides in terms of the control of ovarian cancer *in vitro* and *in vivo*. We carried out a series of experiments to explore whether such a peptide was effective in controlling ovarian cancer growth *in vitro* and *in vivo*. To the best of our knowledge, this study is the first report of such an approach to identify novel targets for ovarian cancer.

## Methods

### Cell lines and culture

A human ovarian cancer cell line, HO8910, was obtained from the Laboratory Animal Center, Sun-Yatsen University, Guangdong, China, and grown in Roswell Park Memorial Institute and cultured in 1640 medium (Invitrogen, Carlsbad, CA,, USA), supplemented with 10 % fetal bovine serum (Hyclone, Logan, Utah, USA). The human ovarian epithelial cell line OSE was obtained from Xiandu Company (Guangzhou, China) and cultured in serum-free DMEM-F12 medium (Invitrogen) containing 10 mg/ml epidermal growth factor (Sigma, St Louis, MO, USA) according to a previously reported method [22]. These cells were incubated at 37 °C in a humidified atmosphere with 5 % CO_2_.

### Phage libraries and biopanning

The Ph.D.-c7c phage display peptide library was purchased from New England Biolabs (Ipswich, MA, USA). This library contained approximately 1 × 10^13^ pfu/mL phages with a diversity of 1.28 × 10^9^ unique peptide sequences for up to 70 copies of each. Biopanning is an affinity selection technique for identifying peptides that bind to a given target [23]. In this study, the biopanning began with the incubation of the phage display peptide library with both normal and tumor cells, in which normal cells were used to deplete peptides that only bind to normal cells and then further incubated with tumor cells for identifying the peptides that only specifically bind to tumor cells. Briefly, 1 ml of the primary phage display peptide library (1 × 10^11^ pfu/ml) in 5 ml of phosphate buffered saline (PBS) was added to the confluent cell monolayer (normal ovarian epithelial cells, called depletor) in a 60 mm diameter dish and the cells were then incubated for 2 h on a rocker platform at 4 °C. After that, the cell culture medium containing unbound phage was collected and transferred to another cell monolayer (HO8910 cells, called tester) and cells were incubated for another 2 h at 4 °C. The cells were then intensively washed four times with Tris-based saline (TBS)-0.2 %. Tween-20 and cell-bound phages were eluted from the cell surface by incubation with 1 mll of elution buffer [0.2 M Glycine-HCL pH 2.2] for 10 min at 4 °C. The elution was immediately neutralized by the addition of 150 ml of 1 M Tris–HCl buffer (pH 9.0) and the aliquot of the eluted phage was used for microtitration, while the remaining phage was subjected to the next rounds of biopanning according to Wang JJ et al. [24]. for a total of four rounds of *in vitro* biopanning. At the end of each biopanning, the ratio of output *vs.* input phage numbers (number of cell-associated phage divided by the numbers of total phage applied to the cells) was calculated for each round.

### Enzyme-linked immunosorbent assay (ELISA)

To identify phage clones, we performed an ELISA assay. Briefly, after four rounds of *in vitro* biopanning, twenty blue plaques were randomly chosen from the titration plate. HO8910 cells were plated into 96-well plates at a density of 10^4^ cells/well, washed and cultured in serum-free medium at 37 °C for 1 h, and finally fixed in 4 % paraformaldehyde for 20 min at room temperature. Cells were then washed three times with PBS-Tween 20 (PBS with 0.05 % v/v Tween 20) and blocked with a blocking buffer (PBST containing 3 % w/v BSA) for 1 h. The selected phage clones (10^10^ pfu/well) and the negative control, M13KE phage (New England Biolabs) were added individually onto the cells and further incubated at 37 °C for 1 h. After that, the cells were washed three times with PBST and then cultured for 1 h in the presence of the HRP-conjugated anti-M13 antibody (Abcam, Cambridge, UK) at a dilution of 1:20 in the blocking buffer. The cells were then added to the Tetramethylbenzidine (TMB) working substrate solution (50 μL/well; Sigma) and incubated for 20 min at room temperature. The incubation was stopped by adding 4 mol/L H_2_SO_4_. Finally, the absorbance was measured at 450 nm using a microplate reader (Bio-Rad model 550, Hercules, CA).

### Immunofluorescence staining

Immunofluorescence staining was performed as described previously [25] to identify positive phage clones that bind to the cell surface. Briefly, after the cells were incubated with phage clones for 1 h at room temperature, the cells were washed with PBS and incubated with M13 antibodies at a dilution of 1:300 (Abcam) for 1 h at room temperature, Cells were subsequently washed with PBST and the second antibodies M13-FITC (Abcam) were added and incubated for 1 h at room temperature. The cells were finally observed using an inverted microscope, equipped with a digital camera and processed using the Viewfinder program. For each experiment, normal human ovarian epithelial cells were used as the negative control.

### DNA sequencing and peptide synthesis

After four rounds of *in vitro* biopanning, twenty blue plaques were randomly chosen from the titration plate and amplified. Their ssDNA were extracted according to the instruction manual (Bioteke, Beijing, China). DNA sequencing analysis was performed by Shanghai Biotechnology (Shanghai, China) and the sequence data were analyzed by using the BLAST and PMOTIF programs. The candidate *Peptide 1* (SWQIGGN, translated from the selected M13 phage DNA sequence) and an irrelevant control peptide (QFHFDAP) were synthesized and labeled with biotin by Shanghai Biotech Bioscience and Technology (Shanghai, China).

### Cell viability assay

The *in vitro* effects of the selected peptides on cells were assessed using the MTT cell viability assay. Briefly, ovarian cancer HO8910 cells were plated into 96-well plates at a density of 10^4^ cells/well, in triplicate and grown overnight. The next day, 10 mM of synthetic peptides were added into the cell culture wells at a final volume of 200 ml of the regular growth medium/well. The irrelevant peptides were used as negative controls. At the end of each time point of the experiment, the media was removed and replaced with 20 ml of 5 mg/ml MTT (3{4,5}-dimethylthia-zol-2,5-diphenyl tetrazolium bromide (Sigma) in the growth medium, and the plates were further incubated in standard conditions for 4 h. Afterwards, the supernatants of the cell culture were removed and replaced with 150 ml dimethyl sulfoxide (DMSO) to solubilize the MTT dye. Absorbance was then determined using a Spectra max 96-plate reader at 490 nm. Ovarian cancer HO8910 cells, without any treatment, were used as the blank group and DMSO was added to the control wells at equal volumes to those used for the test compounds.

### Cell invasion and migration assays

The invasion and motility assays were performed using a Transwell chamber with a 8-μm pore size polycarbonate membrane (Corning, Hangzhou, China) as recommended by the supplier. For the invasion assay, the upper chamber of the polycarbonate filter was coated with 10 μl of Matrigel (New England Biolabs) at a dilution of 1:3 with the growth medium. The chambers were then incubated at 37 °C for 30 min to allow the Matrigel to form a continuous thin layer. The *Peptide 1*-HO8910, control peptide-HO8910, and HO8910 cells were starved for 24 h and then harvested. 5× 10^4^ cells in 200 μl of 0.5 % bovine serum albumin (Sigma) were placed in the upper chamber. The lower chamber was then filled with 600 μl of growth medium subsidized with 20 % fetal bovine serum. Cells were then cultured at 37 °C with 5 % CO_2_ for 48 h. Afterwards, cells on the upper surface of the filter were removed using a cotton swab, whereas cells that invaded through the Matrigel filter to the lower surface were fixed with 4 % neutral-buffered formalin and stained in 0.01 % crystal violet solution. The cell numbers in five fields (up, down, median, left, and right. ×200) were counted for each chamber, and the results were expressed as the mean ± SD. The tumor cell migration assay was performed in the same manner, with the exception that the filter was not coated with Matrigel.

### Cell adhesion assay

To assess cell adhesion capacity after treatment with or without the peptides, we performed a cell adhesion assay. Briefly, the 96-well plate was first coated with 30 μl of Matrigel at a dilution of 1:3 in PBS and incubated overnight with 0.1 % BSA. Three groups of cells (the *Peptide 1*-HO8910, control peptide-HO8910, and HO8910 cells) were washed three times in serum-free medium, resuspended at a concentration of 1 × 10^6 cells^/ml in serum-free medium and added into each well at 100 μl/well and the cells were then incubated at 37 °C for 1 h. Unattached cells were washed away with PBS. 10 μl of the MTT solution was then added into each well and the plates were further incubated for 4 h. 100 μl of DMSO was added to replace the growth medium to dissolve formazan by shaking for 10 min. Absorbance values (A) were measured at a wavelength of 492 nm using a microplate reader. Results were expressed as the mean ± SD and the adhesion rate was calculated using the formula: Relative adhesion rate (%) = (A_492_ of experimental group/A_492_ of control group) × 100 %. Each experiment was repeated three times.

**Table 1:** Specific enrichment of HO8910 cell-bound phages using an initial input of 10^11^ pfu

Round	Inputs (pfu/ml)	Outputs (pfu/ml)	Recovery rate (%)
1	2 × 10^11^	0.9 × 10^5^	4.5 × 10^−6^
2	2 × 10^11^	3.4 × 10^6^	1.7 × 10^−5^
3	2 × 10^11^	4.4 × 10^7^	2.2 × 10^−4^
4	2 × 10^11^	2.2 × 10^8^	1.1 × 10^−3^

**Fig. 1:**
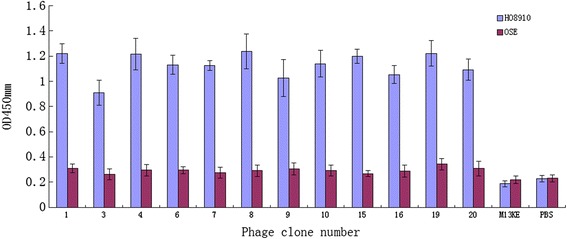
Efficacy of phage clones in binding to HO8910 versus normal ovarian cells. HO8910 cells and OSE were incubated with phage display peptides. Unbound phages were removed by gentle washing and the bound phage clones were assayed by ELISA after incubation with HRP/anti-M13 and then analyzed with a microplate reader at 450 nm. The mean OD values represent activity of phage binding to cells. M13KE and PBS were used as controls. Lanes 1 to 12 depicts positive phage clones biopanned with HO8910 versus OSE cells

### Animal studies

Twenty-one female athymic BALBC/c nude mice (4 to 6 weeks old with weights of 14.96 ± 0.96 g; animal approval number: 0113061) were obtained from Guangdong Medical Laboratory Animal Center, Guangzhou, China (SCXK(YUE)2008-0002) and maintained in Guangzhou Medical College Laboratory Animal Center, and were kept in specific pathogen free, temperature-controlled isolation conditions, and fed with sterilized food and autoclaved water. Animal breeding, care, and all experimental procedures were approved by the ethical and humane committee of Guangzhou Medical College and carried out strictly in accordance with the related regulations on administration of experimental animals under the SPF animal experiment number: 0052246. Specifically, these 21 mice were randomly divided into three groups: HO8910 cells pre-incubated with *Peptide 1*, HO8910 cells pre-incubated with the control peptide, and HO8910 cells without pre-incubations. Cells were then injected into the enterocoelia of each nude mouse with 1 × 10^6^ cells in 200 ml volume. The mice were then observed every two days and the abdominal circumference was measured. On day 42, the mice were euthanized and the tumors were dissected after the abdominal circumference and ascitic volume were measured. The tumor disseminated localization and the number of individual disseminated tumors were counted and the inhibitory rate was then calculated as follows: (control group average tumor weight - the average tumor weight)/control group average tumor weight] × 100 %. Tumor tissues were assessed for histopathology, immunohistochemistry and apoptosis.

### Immunohistochemistry

To detect expression of vascular endothelial growth factor (VEGF) in tumor tissues from animal experiments, we performed immunohistochemistry using a rabbit polyclonal anti-VEGF antibody (Sigma, St Louis, MO, USA) according to the manufacturer’s instructions. The anti-VEGF antibody was diluted to 1:100 and the biotinylated goat anti-rabbit IgG was diluted to 1:200. The staining results were evaluated by two independent investigators who were unaware of the identity of the tissue samples.

### Terminal deoxynucleotidyl transferase dUTP nick end labeling (TUNEL) assay

TUNEL assay was used to detect apoptosis levels in tumor tissues using an *in situ* cell death detection kit conjugated with horseradish peroxidase (POD) (Roche Applied Science, Indianapolis, IN, USA) according to the manufacturer’s instructions. The rate of apoptosis was evaluated by counting TUNEL-positive cells (brown-stained) and the apoptotic index was defined as the number of TUNEL-positive cells/total number of cells in 5 randomly selected high-power fields (magnification × 400).

**Table 2:** Peptide identified for HO8910 cell binding (Amino Acid Sequences, deduced from DNA)

Clone	Peptide sequence	Incidence
Phage 1, 3, 7, 16, 19, and 20	SWQIGGN	6
Phage 4	QFHFDAP	1
Phage 6	TSPFVVP	1
Phage 8	TGNSNTQ	1
Phage15	TSHFEVP	1
Phage 9 and 10	IGNSNTL	2

### Statistical analysis

Statistical analysis was performed with SPSS 13.0 statistical software (SPSS Inc., Chicago, IL, USA). Results were expressed as the mean ± SE of three or more observations (as indicated in each experiment). Differences amongst the three groups were assessed using one-way ANOVA and differences between the two groups were assessed using the Student-Newman-Keuls (SNK). A *p* value equal to or less than 0.05 was considered statistically significant.

**Fig. 2:**
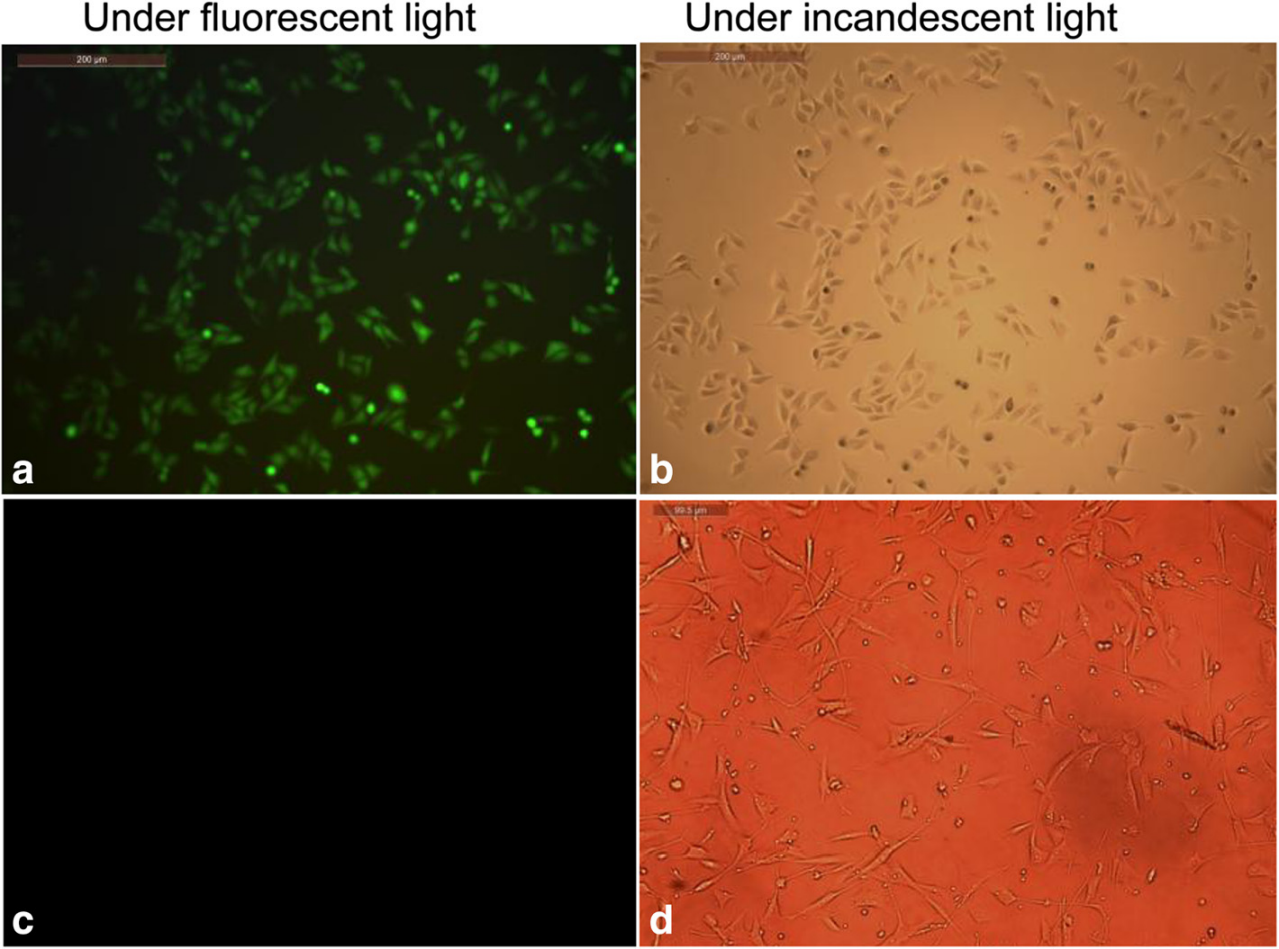
Binding of Peptide 1 to the cell surface (×280). Immunofluorescence was used to stain Peptide 1 binding to HO8910 cells. Cells were visualized using a Leica DMRA2 fluorescence microscope. **a** and **b** HO8910 cells. **c** and **d** OSE cells

## Results and discussion

### Identification of phage peptides that specifically bind to the surface of ovarian cancer cells

In this study, we first screened and identified peptides that could specifically bind to the cell surface of ovarian cancer cells using the phage display assay. The input/output phage ratio was calculated for each round of HO8910 cell biopanning and the ratio gradually rose and tended to stabilize after the fourth round. The data showed that the number of the fourth round phages were 244 times higher than that of the first round experiment (Table 1). After that, we performed ELISA to confirm the cell binding affinity of twenty selected phage clones. As shown in Fig. 1, 12 phage clones had very high affinity to bind to HO8910 cells compared to that of OSE cells. Thus, these 12 positive phage clones were propagated and ssDNA was extracted for DNA sequencing. After that, we identified unique DNA sequences that contained seven amino acids (Table 2). After similar peptide sequences had been aligned, most of the sequences revealed a consensus sequence at their amino terminal end and the peptide SWQIGGN appeared more frequently and then the peptides were blasted using the BLAST and PMOT IF programs to the Genbank database. However, there was no homology to any known protein that contained the phage peptide SWQIGGN, and thus, we named this clone Peptide 1.

**Fig. 3:**
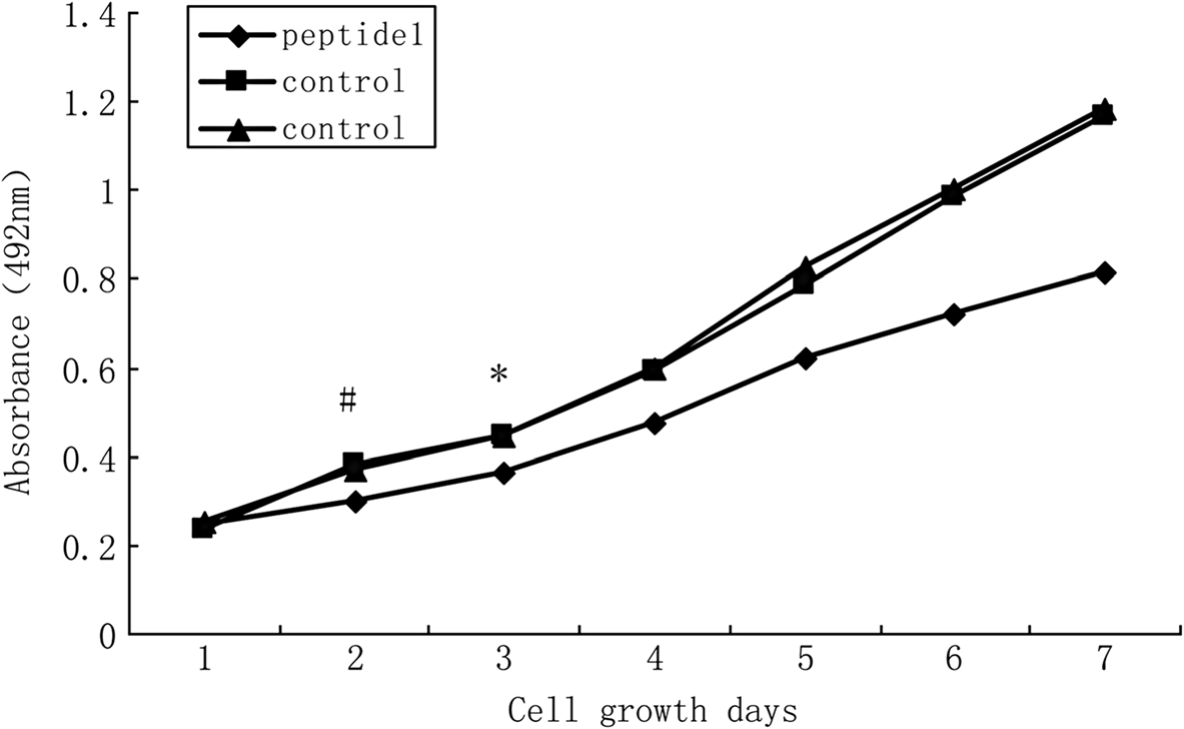
Effects of *Peptide 1* on the regulation of HO8910 cell viability. Ovarian cancer cells were treated with Peptide 1, negative control peptide, and blank control for up to 7 days and cell viability was assessed using a MTT assay. *P <0 .05

We subsequently performed immunochemistry tests to further confirm the binding of Peptide 1 to the HO8910 cells. As shown in Fig. 2, Peptide 1 was able to bind to HO8910 cells, but not normal human ovarian epithelial cells. Moreover, the negative control peptide clones did not significantly bind to both of the cell lines. These data indicate that Peptide 1 (i.e., SWQIGGN) was able to specifically bind to HO8910 cells.

**Fig. 4:**
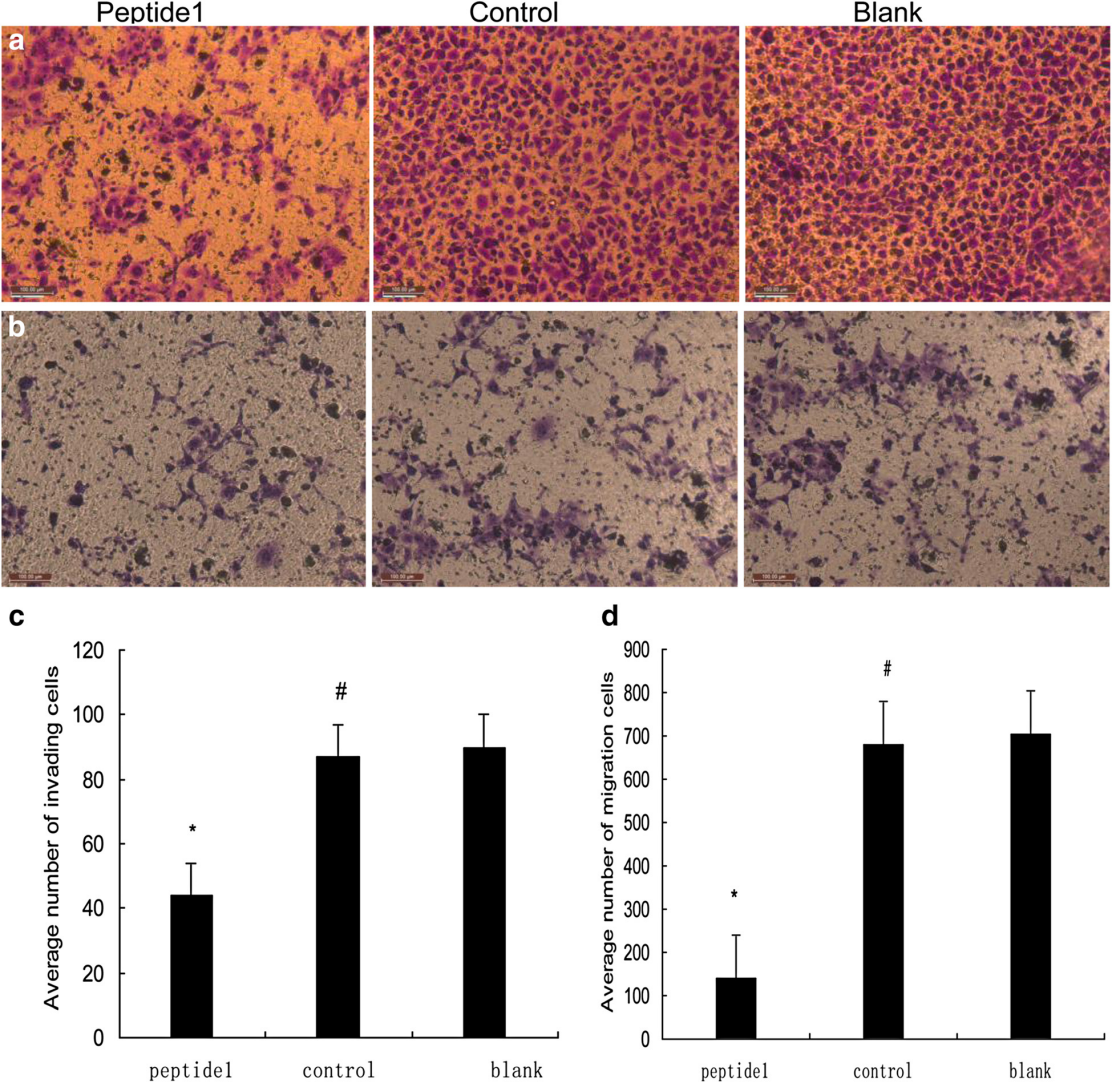
Effects of Peptide 1 on the regulation of ovarian cancer HO8910 cell metastatic phenotypes. **a** Matrigel invasion assay; **b** Transwell tumor cell migration assay. The number of invading and migrating tumor cells was determined by counting the cells stained with 0.01 % crystal violet solution in the lower side of Transwell filter. The decrease in the number of invading and migrating cells in Peptide 1-treated tumor cells compared to those of the parental and negative control peptide cells (*P < 0.05). **c** and **d** represent quantitative data from **a** and **b**

### Effects of selected Peptide 1 on the regulation of ovarian cancer HO8910 cell biology *in vitro*

We then assessed the effect of Peptide 1 on the regulation of ovarian cancer cell viability, apoptosis, invasion, and adhesion *in vitro*. As shown in Fig. 3, there were no differences in cell viability among the three treated groups at day 1 and 2 (*P* > 0.05); however, between day 3 and 6, Peptide 1 significantly reduced ovarian cancer cell viability (*p* < 0.05), but there were no differences in cell viability between the VCSM13-HO8910 and the blank controls (*p* > 0.05).

Furthermore, the tumor cell Transwell migration and invasion assay showed that HO8910-Peptide 1 had a pronounced reduction in both cell migration and invasion capacity compared to the negative peptide-treated and blank control cells (*p* < 0.05), whereas the negative peptide-treated and blank control cells showed similar migration and invasion capacity (*p* > 0.05; Fig. 4). These results indicated that Peptide 1 was able to block the metastatic potential of HO8910 cells *in vitro*.

**Fig. 5:**
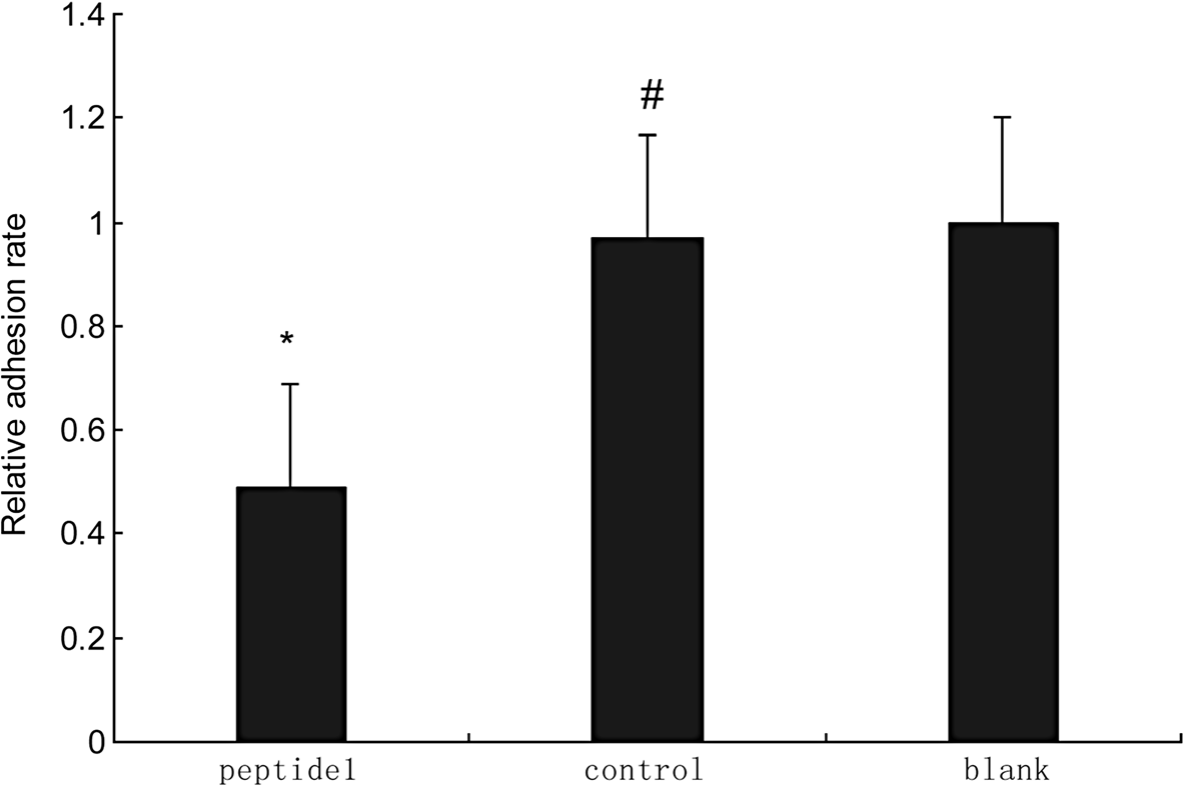
Effect of Peptide 1 treatment on regulation of ovarian cancer cell adhesion capability. Ovarian cancer cells were treated with Peptide 1, negative control peptide, and blank control for five days and then the cells were seeded onto Matrigel-coated cell culture dishes and grown for 1 h. Cells were washed with PBS and the remaining cells were visualized using MTT staining and quantified. *P < 0.05 compared to the negative control peptide-treated cells and blank cells; #*P* > 0.05 versus blank cells

In addition, we also assessed the effect of Peptide 1 on the regulation of HO8910 cell adhesion and found that Peptide 1 significantly blocked HO8910 cell adhesion capacity compared to the negative peptide-treated or bank control cells (*p* < 0.05), whereas the negative peptide-treated and blank control cells showed similar potential in terms of adhesion capacity (*p* > 0.05; Fig. 5).

### Effects of selected Peptide 1 on ovarian cancer HO8910 cells *in vivo*

**Fig. 6:**
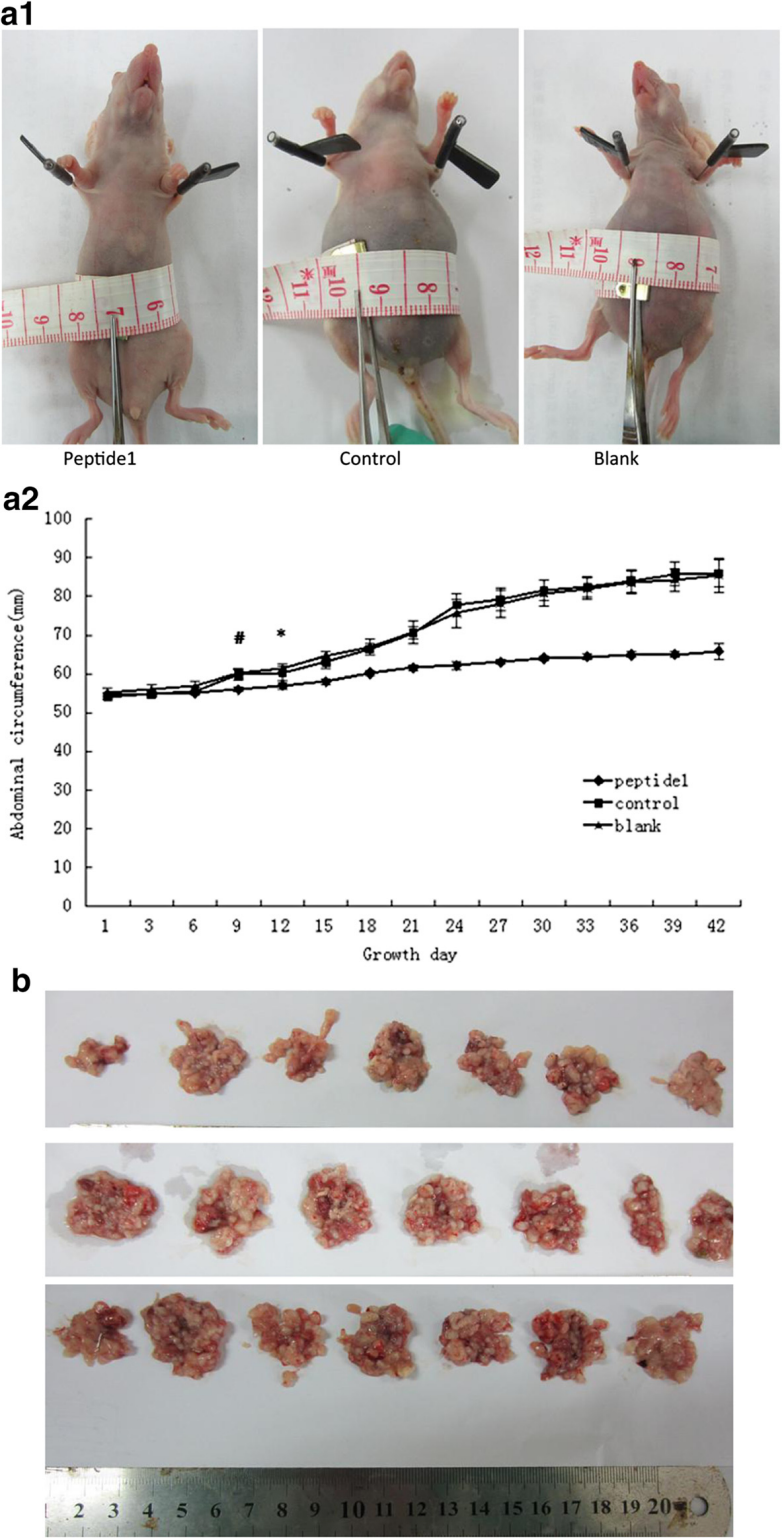
Effect of Peptide 1 treatment on regulation of ovarian cancer cell growth in nude mice in an intraperitoneal tumorigenicity model. Comparison of abdominal circumferences of three groups of nude mice. **a1** Abdominal circumference in Peptide 1 group versus negative control peptide and blank controls (*p* < 0.05). **a2** Abdominal circumference growth curves of Peptide 1, negative control peptide, and blank control mice (*P < 0.05 from this point onwards). **b** Comparison of tumor growth in three groups. Data are presented as mean ± SD. *P < 0.05 versus the negative control peptide and Blank; #*P* >0.05 versus the Blank

To further investigate whether Peptide 1 can control ovarian cancer *in vivo*, we treated ovarian cancer cells with Peptide 1 or negative control peptides for five days and then injected them into the enterocoelia of each nude mouse. As shown in Fig. 6, the abdominal circumference and mouse body weights were similar at the beginning of the experiment, but the abdominal circumference of HO8910-Peptide 1 cell-bearing mice didn’t show much change two weeks after tumor cell injection, i.e., 65.8 ± 2.1 *mm*, whereas the abdominal circumference of mice bearing HO8910-negative control peptide-treated and HO8910 parental cells was 86.0 ± 3.4 *mm* and 85.4 ± 4.2 *mm*, respectively (*P* < 0.05). As shown in Table 3, the volume of ascites, the number of tumors, the total number of disseminated tumor nodules and the tumor weight were all significantly smaller in the Peptide 1 treated cell group than those of the HO8910-negative control peptide and HO8910 parental cell groups (*p* < 0.05). However, no statistical significance in tumor growth was observed between control and HO8910 groups (*p* > 0.05). These results suggested that Peptide 1 was able to inhibit the growth and dissemination of ovarian cancer cells in this mouse transplantation model.

### Effects of Peptide 1 on the regulation of VEGF expression and the apoptosis of ovarian cancer HO8910 cells in transplanted tumor tissues in mice

**Fig. 7:**
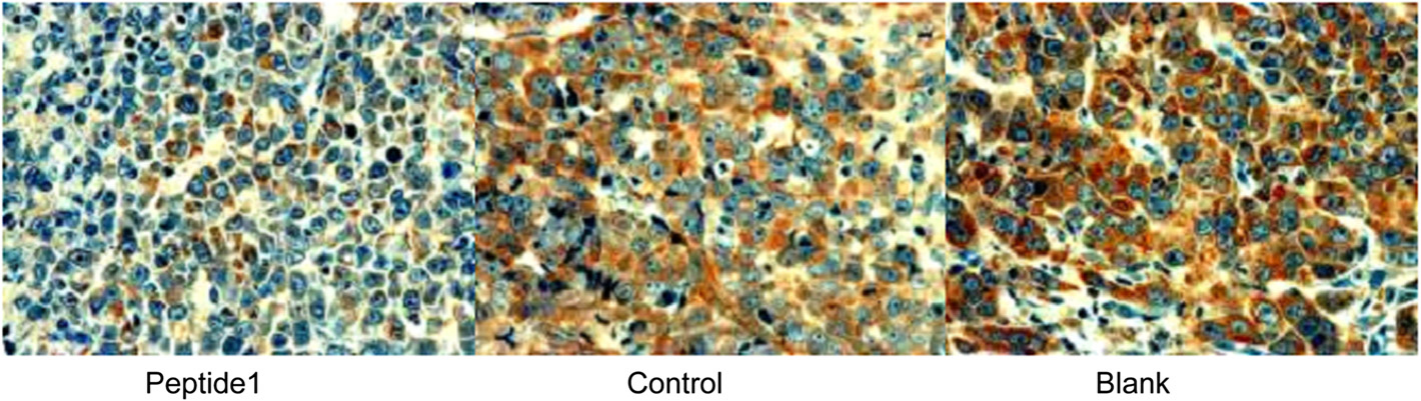
Effect of Peptide 1 treatment on regulation of VEGF expression in nude mice in an intraperitoneal tumorigenicity model (×400). Mouse tumor tissues were dissected and immunostained with anti-VEGF antibody. Stained tissue sections were reviewed and scored (see Methods section). Expression of VEGF in mouse tumor tissues of Peptide 1-treated tumor cells was markedly decreased compared to mouse tumor tissues of the negative control peptide-treated tumor cells and parental HO8910 cells

To further assess the effects of Peptide 1 on ovarian cancer cells *in vivo*, we analyzed the expression of VEGF protein in ovarian cancer HO8910 cell transplanted tumor tissues in mice. As shown in Fig. 7, immunohistochemical staining data showed that the expression of VEGF protein in Peptide 1 treated ovarian cancer cells was markedly lower than in the control HO8910 cell group (*p* < 0.05), indicating that *Peptide 1* could inhibit the expression of VEGF in HO8910 cells *in vivo*.

**Table 3:** Effect of peptide 1 on malignant behaviors of ovarian cancer xenografts in nude mice

Group	TFN	MTF	MR (%)	TV (ml)	TW (g)	IR (%)
Blank	7 (7/7)	103 ± 26.40	100	1.40 ± 0.64	1.77 ± 0.44	_
Control	7 (7/7)	94.86 ± 30.62	100	1.32 ± 0.75	1.67 ± 0.37	5.65
*Peptide 1*	7 (5/7)	28.60 ± 13.16^*^	71.4^*^	0.24 ± 0.18^#^	0.52 ± 0.28*	70.62

*TFN* tumor formation number, *MTF* metastatic tumor foci, *MR* metastatic rate, *TV* tumor volume, *TW* tumor weight, *IR* inhibitory rate; The values of metastatic tumor foci and tumor weight are presented as mean ± SD of *n* = 7 mice in relevant groups. *P < 0.001 and ^#^ *p* < 0.05 vs. the control and HO8910 groups

*TFN*, Five weeks post-inoculation, the nude mice were sacrificed, and then tumor formation was calculated for each mouse

*MTF*, collection of all tumors in each mouse, and then measurements of the number of tumors

*MR*, the MR of control = the MIF of control/ the MIF of blank; the MR of *Peptide 1* = the MIF of *Peptide 1* /the MIF of blank;

*TW*, collect all the tumors from each mouse, and then their weights by scale

*IR*, IR of control = [1-(the weight of control/the weight of blank)] *100 %

IR of *Peptide 1* = [1-(the weight of *Peptide 1*/the weight of blank)] *100 %

### Peptide 1 promoted cell apoptosis *in vivo*

**Fig. 8:**
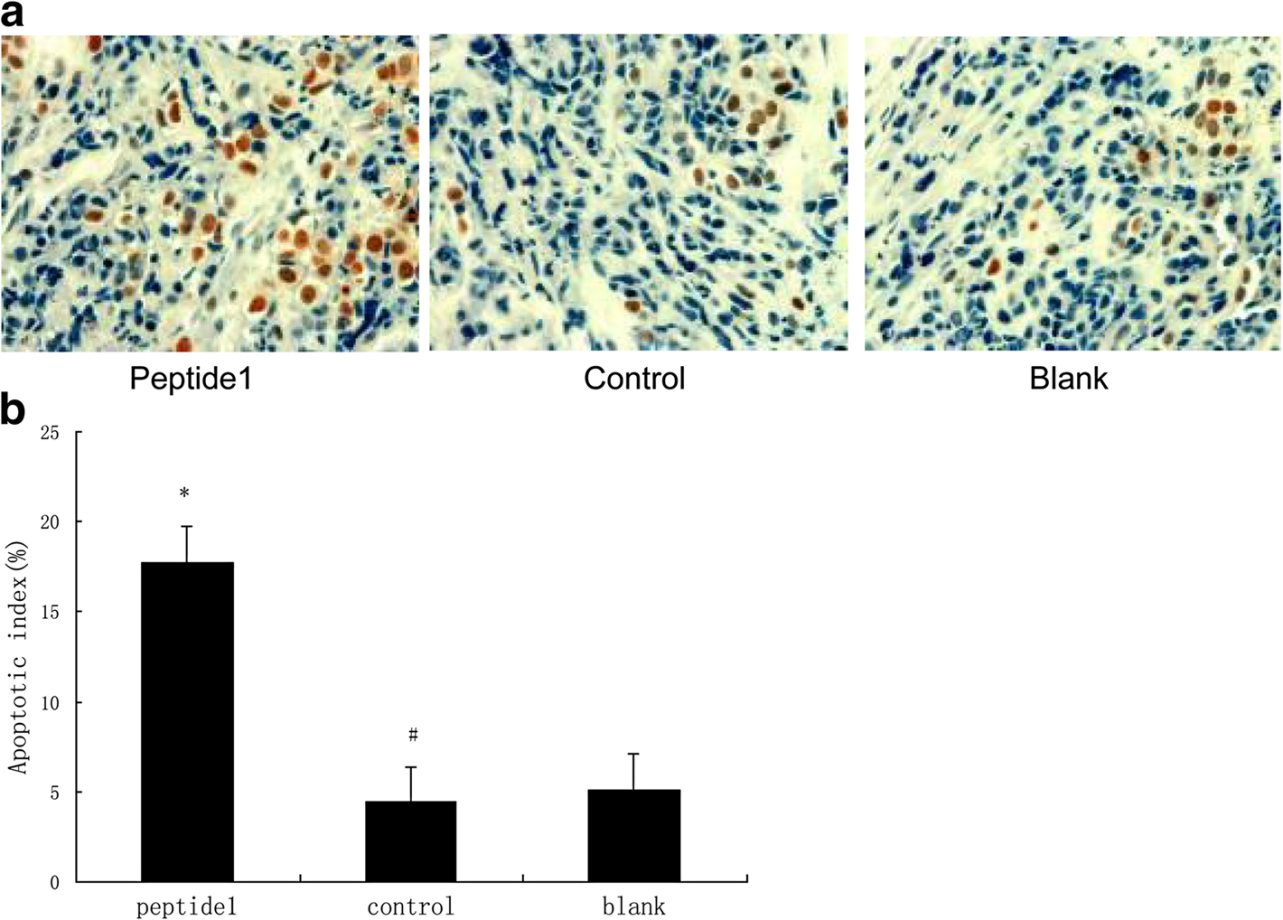
Effect of Peptide 1 treatment on induction of apoptosis in a nude mouse intraperitoneal tumorigenicity model (×400). Mouse tumor tissues were dissected and immunostained for TUNEL assay. Stained tissue sections were reviewed and scored (see Method section). **a** Representative images of tumor sections examined by TUNEL assay. **b** The apoptotic index significantly increased in mouse tumor tissues of Peptide 1 group tumor sections compared to those of the negative control peptide and parental group tumor sections. The data are presented as the average number of apoptotic index ± SD. *P < 0.05 versus the negative control peptide and parental group; #*P* > 0.05 versus parental group

We then performed the TUNEL assay to detect the effect of Peptide 1 on the induction of cell apoptosis in tissue specimens from the nude mouse intraperitoneal tumorigenicity model. As shown in Fig. 8, the TUNEL-positive nuclei were stained brown, and the apoptotic index was significantly higher in the Peptide 1 group compared to the control or HO8910 group (*p* < 0.05). However, there was no statistical significance observed between the control and HO8910 groups (*P* > 0.05). These results indicated that Peptide 1 promoted cell apoptosis *in vivo*.

Novel approaches could be used to identify and develop more effective therapeutic agents for the control of ovarian cancer in clinic. In this study, we utilized the phage display technique to screen and identify peptides that specifically bind to ovarian cancer cells to act as a therapeutic strategy. We identified 12 phage clones that could specifically bind to ovarian cancer cells but not normal ovarian epithelial cells. After DNA sequencing and blast search of Genbank, we found a novel peptide SWQIGGN, which did not have an homology to any known protein. We then confirmed its ability to bind to ovarian cancer cells immunochemically and we explored the effects of this peptide on the control of ovarian cancer *in vitro* and *in vivo.* Our data showed that the treatment of ovarian cancer cells with this peptide significantly reduced tumor cell viability and suppressed tumor cell migration, invasion, and adhesion capacity. *In vivo* nude mouse studies showed that peptide treated-ovarian cancer cells had much smaller ascite volume, a smaller number of disseminated tumor nodules, and smaller tumor total weights than those of negative control peptide-treated HO8910 (*P* =0.0035) and parental HO8910 (*P* =0.0087) cells. Treatment with this peptide also inhibited VEGF expression. Thus, our current data identified a phage display-identified tumor cell-binding peptide that was able to control ovarian cancer cell viability, migration, invasion, adhesion capacity i*n vitro* and tumor growth and metastasis *in vivo*. Future studies are needed to evaluate its efficacy and side effect profiles before it can be translated into clinical applications for the treatment of ovarian cancer patients.

The phage display technique is a useful tool to identify short peptides or antibodies that can specifically bind to and regulate functions of target proteins and the latter could be used for early tumor diagnosis, vaccines and as therapeutic targets. If proteins are expressed on the cell surface, they could also be useful for the delivery of bioactive agents, such as small-molecule drugs and radionucleotides to specifically target the cells which express these proteins [26]. Recently, this technology was utilized to screen prostate and breast cancer cell-specific functional peptide ligands [27]. Our study identified a group of ovarian cancer cell-specific binding peptides and we chose one of these peptides for further study as a therapeutic agent for ovarian cancer *in vitro* and *in vivo*. The data demonstrated the usefulness of this technique to screen and identify tumor cell specific ligands.

In this study, we first biopanned our phage libraries with normal ovarian epithelial cells in order to remove the peptides that can bind to normal cells and then biopanned the remaining phage libraries with ovarian cancer cells to identify peptides that specifically bind to ovarian cancer cells. We obtained the ratio of output to input with an enrichment of 244 times in the last round compared to the first round. This study differed from that of other studies [28] in that they reported their data by selected cell surface-bound peptides to specifically bind to ovarian cancer cells but not stratified by normal cells. After that, we selected 20 phage clones for confirmation and 12 of them were shown to specifically bind to tumor cells versus normal cells. DNA sequencing and a blast search of Genbank identified a novel peptide SWQIGGN that didn’t show any homology to any known protein. Thus, this peptide could be useful in the control of ovarian cancer; therefore, we then assessed the effects of this peptide on affecting the biological behaviors of ovarian cancer cells. The data showed that this peptide was able to reduce tumor cell viability, migration, invasion, and adhesion capacity. However, to date, it is unclear how this peptide blocked biological cell behavior. Future studies are needed to identify the protein or cell structure that this peptide binds to. However, since this peptide binds to a surface protein on tumor cells, such a protein could be a cell growth factor or hormone receptor and the binding of this peptide blocks the ligands-binding to the protein and therefore, inhibits their signaling transduction leading to the suppression of tumor cell growth, migration and adhesion capacity. Negative controls (non-specific) peptides used in the current study didn’t show much activity in ovarian cancer cells. This negative control (i.e., IGNSNTL) was obtained after the fourth screening (Table 2).

Moreover, cell motility and tumor cell invasion are major components in the multistage process of cancer metastasis [29, 30]. It is demonstrated that *the in vivo* blockade of tumor cell spreading can suppress tumor cell movement and invasiveness *in vitro*. Our current data showed that this peptide inhibited ovarian cancer cell adhesion, migration, and invasion capacity *in vitro*. Our nude mouse experiment results complimented our *in vitro* data, showing that peptide-treated ovarian cancer cells had smaller tumor numbers and size, and metastasis lesions in nude mice. At the gene level, this peptide inhibited VEGF expression in tumor tissues. Indeed, tumor angiogenesis is the key to facilitate tumor growth and metastasis [2, 31]. However, it is unknown whether this peptide did block tumor angiogenesis by downregulation of VEGF expression. VEGF is the most important vascular growth factor [31] and its expression is regulated by different factors [32]. Thus, future studies will be needed to explore how this peptide reduces VEGF expression.

## Conclusions

Our current study identified a novel peptide (SWQIGGN) that can 1) specifically bind to ovarian cancer cells and 2) control ovarian cancer *in vitro* and *in vivo*. However, our current study is just proof-of-principle and more thorough studies need to be performed before this peptide can be used in a clinical setting. Our future studies will focus on: 1) understanding the underlying mechanism of its action in ovarian cancer cells and identifying binding protein(s), 2) exploring the specificity of this peptide in other ovarian cancer cell lines versus other types of cancer cells and 3) assessing the clinical efficacy and side effects of this peptide in ovarian cancer patients.

## Abbreviations

AI: apoptotic index
Anti-M13: AntiI-M13 monoclonal antibody
BSA: bovine serum albumin
DAB: diaminobenzidine
EDTA: eathylene diamine tetraacetic acid
EGF: epidermal growth factor
ELISA: enzymelinkedimmunosorbent assay
FITC: fluorescein Isothiocyanate
HE: hematoxylin-eosin staining
HOSE: human ovarian surface epithelium
HRP: hoeseradish peroxidase
HRP/Anti-M13: horseradish peroxidase conjugated to anti-M13 monoclonal antibody
MTT: methyl thazoy terazolium
PBS: phosphate buffered saline
SP: streptavidin-perosidase
TUNEL: termina deoxynucleotidyl transferase-mediated dUTP-biotin nick end labeling assay
VEGF: vascular endothelial growth factor
